# Influence of Freezing and Different Drying Methods on Volatile Profiles of Strawberry and Analysis of Volatile Compounds of Strawberry Commercial Jams

**DOI:** 10.3390/molecules26144153

**Published:** 2021-07-08

**Authors:** Doaa Abouelenein, Ahmed M. Mustafa, Simone Angeloni, Germana Borsetta, Sauro Vittori, Filippo Maggi, Gianni Sagratini, Giovanni Caprioli

**Affiliations:** 1 School of Pharmacy, University of Camerino, Via Sant’Agostino 1, 62032 Camerino, Italy; doaa.abouelenein@unicam.it (D.A.); ahmed.mustafa@unicam.it (A.M.M.); simone.angeloni@unicam.it (S.A.); germana.borsetta@unicam.it (G.B.); sauro.vittori@unicam.it (S.V.); filippo.maggi@unicam.it (F.M.); gianni.sagratini@unicam.it (G.S.); 2 Department of Pharmacognosy, Faculty of Pharmacy, Zagazig University, Zagazig 44519, Egypt

**Keywords:** strawberry, aroma, volatile organic compounds, drying methods, freezing, commercial jams, HS-SPME/GC-MS

## Abstract

Strawberry is the most consumed berry fruit worldwide due to its unique aroma and flavor. Drying fruits to produce a powder represents one of the possible conservation methods to extend their shelf-life. The aim of the present study was to compare the influence of freezing and different drying methods on the volatile profile of strawberry using the HS-SPME/GC–MS method, in addition to analysis of strawberry jam volatiles. A total of 165 compounds were identified, accounting for 85.03–96.88% of the total volatile compositions. Results and PCA showed that freezing and each drying process affected the volatile profile in a different way, and the most remarkable representative differential volatiles were ethyl hexanoate, hexyl acetate, (*E*)-2-hexenyl acetate, mesifurane, (*E*)-nerolidol, *γ*-decalactone, 1-hexanol, and acetoin. Shade air-dried, frozen, freeze-dried, and oven-dried 45 °C samples retained more of the fruity and sweet aromas of strawberry, representing more than 68% of the total aroma intensity according to the literature. In contrast, the microwave-drying method showed drastic loss of fruity esters. Strawberry jams demonstrated complete destruction of esters and alcohols in most jams, while terpenes were significantly increased. These findings help better understand the aroma of strawberry and provide a guide for the effects of drying, freezing, and jam processing.

## 1. Introduction

Berries constitute a large group of functional foods, known as “superfoods”, whose consumption delivers several health benefits beyond basic nutrition [1]. Strawberry is one of the most demanded berries in fresh and frozen forms, as well as in processed and derived food products such as jam [2]. The aroma of strawberry fruits has received an increasing amount of attention from both producers and consumers due to the perceived importance of the sensory quality of commercially produced cultivars.

Strawberries (*Fragaria* × *ananassa* (Duchesne ex Weston) Duchesne ex Rozier) are the most commonly used berry fruits worldwide, and they are characterized by unique aroma and flavor [3,4]. Due to the highly fragile structure of strawberries, their postharvest life is relatively short. The perishability and inherent short life of the fruit can result in rapid changes in the volatile compound profile [5]. However, the volatile organic compounds are responsible for the volatile quality attributes and attraction of customers, and they are closely linked to the general liking of strawberry fruits [6]. Volatile compounds are significant components of strawberry flavor, and slight changes may significantly modify the taste. The aroma of strawberry is considered complex. To date, more than 360 volatile chemicals have been observed in fresh strawberry fruits. These include esters, alcohols, ketones, furans, terpenes, aldehydes, and sulfur compounds [3]. Types and concentrations of volatiles contributing to strawberry aroma vary according to their cultivar and maturity [7]. Although furanones, sulfur compounds, terpenoids, and some other compounds exhibit much lower quantities compared with esters, these compounds demonstrated significant effects on strawberry odor perception [3].

Drying processes occupy an important place in the food industry. Fresh strawberry fruits are perishable and rapidly deteriorate, leading to great economic losses. In addition, their harvest seasons are brief and, therefore, the fruits are not available all year round, which limits their commercialization and consumption. In order to take advantage of the nutritional benefits of strawberry, drying the fruits to produce a powder represents one of the possible conservation methods to extend shelf-life, as well as potentially increase the use of the fruit [8]. It is possible for dried fruits or foods to be easily stored for long periods, due to a decrease in water content, which can also help in the production of new functional food products. The dried fruits can be used in food formulations, and this approach has great importance for consumers and the food industry. In fact, recent studies have reported the application of fruit powders as functional ingredients in foods such as desserts, food concentrates, dietary supplements, and fruit teas [8]. Different drying methods are viable options for production of strawberry powders, including oven-drying, natural air-drying, microwave-drying, and freeze-drying. Oven-drying is a commonly used and cheap method; however, it offers exposure to high temperatures and oxygen, which may change the chemical composition of fruits. In addition, freeze-drying is considered as one of the best drying methods for the production of high-quality fruit powders, but it is quite expensive. Nevertheless, although freeze-drying preserves sensory attributes, some authors have reported that it might lead to a loss or change of bioactive compounds [9,10].

To the best of our knowledge, the effect of different drying methods on the volatile profile of strawberry fruits has been scarcely investigated [11,12,13]. Therefore, the main and first aim of the present study was to compare the influence of different drying methods (oven-drying at 45 °C and 60 °C, freeze-drying, microwave-drying, shade air-drying) for the production of strawberry powders with desirable aroma, which is one of the most important quality attributes, using a new HS-SPME/GC-MS method. This is the first time that HS-SPME/GC-MS has been used for such a comparative study and the determination of differences in the volatile profiles of strawberry fruits, considering the above drying methods and the freezing process. The produced powders can be used in the future as functional ingredients for the development of food products. The experiments were, therefore, designed to test whether the volatile profile of dried and frozen strawberries differed from that of fresh berries. Furthermore, consumption of commercial jams has become more and more popular every day due to the modern lifestyles that consumers follow. Therefore, it is critical for the food industry that the commercial jam products meet consumer satisfaction in terms of their taste or flavor, aroma, texture, and appearance, in addition to the potential health benefits. Few articles have dealt with strawberry jam and the utilization of volatile fraction analysis to detect the adulteration of soft fruit purees using SPME-GC [14,15,16,17]. Accordingly, one of the main motivations for this study was the limited research available in the literature on volatile compounds of the commercial jams of strawberry sold on the market. Therefore, an additional goal of this work was to analyze the volatile profiles of some of the most common commercial strawberry jams by applying HS-SPME/GC-MS, to evaluate the originality of the jam, as well as to estimate the effect of manufacturing and processing, to establish whether the aroma compounds were still at satisfactory levels in commercial jams.

## 2. Results and Discussion

### 2.1. Optimization of HS-SPME

The effects of the main SPME variables such as extraction time (15 and 30 min), extraction temperature (60, and 80 °C), and fiber type (DVB/CWR/PDMS 80 μm, and PDMS/DVB 65 μm) were evaluated, in order to determine the optimum HS-SPME conditions for profiling volatiles in strawberry samples. Results showed that the optimum extraction temperature and time were 60 °C and 30 min, respectively. The DVB/CWR/PDMS fiber seemed to be optimal because it gave the best results in terms of number and peak areas of the detected volatiles in the strawberry matrix. The addition of NaCl solution (25%) increased the intensity of a few peaks, but decreased others; thus, it was better to work without the addition of NaCl salt. Therefore, 30 min extraction time and 60 °C extraction temperature, in addition to 20 min equilibrium time using DVB/CWR/PDMS fiber, were chosen as the optimum HS-SPME conditions. Taking into account the robustness of the method and repeatability of results, these optimized SPME parameters were selected for further investigations of analysis and comparison of the volatile fractions from dried and frozen strawberry fruits and their commercial jam with respect to fresh ones.

### 2.2. Application of the HS-SPME/GC-MS Method to Strawberry Fruit Samples

Food aroma is an important parameter to evaluate the sensory quality of fruit products and fruits. Fruit aroma production is dependent on many factors, such as cultivar, maturity, and storage conditions [7]. Aroma is characterized by a complex composition because of the presence of a wide variety of volatiles with different chemical and sensory nature. These volatile chemical compounds fall into several categories, including esters, terpenes, aldehydes, alcohols, and furanones, which are considered to be the most important aroma perception indicators for fresh fruits [18]. Particularly in commercial strawberry fruit, only 15 odor-active compounds contribute to the strawberry flavor [19], with the esters being the most important group [20]. 

SPME analysis of berries is a good method to understand the volatile composition of the intact fruit, and it decreases the development of byproducts; however, analysis of headspace compounds is dependent upon their individual vapor pressure. Thus, the more volatile analytes might be more easily extracted and appear at comparatively higher concentrations. This reflects the compound’s contribution to the fruit aroma, but does not give its true concentration in the tissue. Thus, volatiles present in the analyzed samples are presented as relative percentage [21,22].

**Figure 1:**
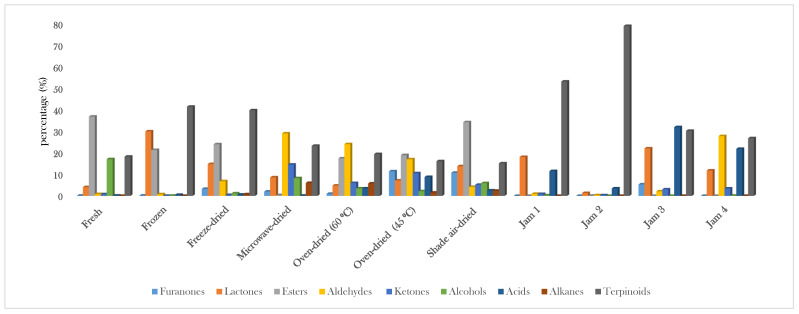
Schematic diagram representing the variations in the major chemical classes detected in different samples of strawberry fruits and jams: Jam 1 (Coop Vivi verde jam); Jam 2 (Coop jam); Jam 3 (Zuegg jam); Jam 4 (Alce Nero jam).

**Figure 2:**
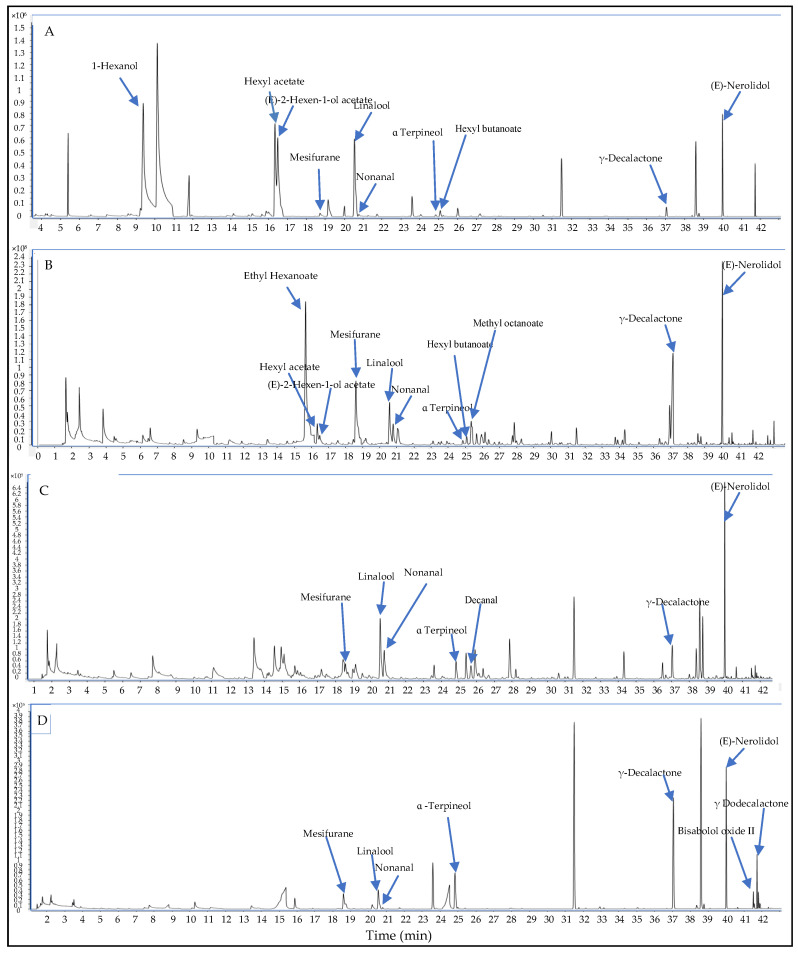
HS-SPME/GC-MS chromatograms of four different samples of strawberry: (**A**) fresh; (**B**) shade air-dried; (**C**) microwave-dried; (**D**) Jam 3.

In this study, our newly developed and optimized HS-SPME/GC-MS method was successfully applied in the analysis of the volatile fraction of 11 samples of fresh, frozen, and dried strawberry fruits and some commercial jams using the selected DVB/CWR/PDMS fiber. A total of 165 volatile compounds were identified in the analyzed samples (Table 1), accounting for 85.03–96.88% of the total headspace composition. The identification of all compounds was based on comparison of retention index (RI) and mass spectra with those reported by NIST 17 (National Institute of Standards and Technology), Adams (2007) and Wiley library spectra [22]. The chemical classes detected in the analysed strawberry samples were esters (52 compounds), terpenes (31 compounds), aldehydes (19 compounds), alcohols (16 compounds), ketones (15 compounds), lactones (eight compounds), furanones (four compounds), and acids (four compounds) (Table 1, Figure 1). Many compounds appear to be primarily responsible for strawberry characteristic flavor such as esters (e.g., methyl butanoate, ethyl butanoate, ethyl hexanoate, methyl butanoate), furanones (2,5-dimethyl-4-hydroxy-3(2*H*)-furanone (DMHF) and its derivative), and terpenoids (e.g., linalol and nerolidol) [3,18,23,24], all of which were detected in our samples. According to the differences in volatile composition between strawberry powders and frozen samples, it was possible to infer that each drying and freezing process affected volatile composition in a different way, and some chromatograms are reported in Figure 2. The variation in ester content, as well as that of terpenoids, furanones, lactones, alcohols, and others, is listed in Table 1. Moreover, the influence of drying treatments (oven-drying at 45 °C and 60 °C, freeze-drying, microwave-drying, or shade air-drying) and freezing on the volatile profile of strawberry fruits with respect to fresh fruits (control) and a comparison of their detected aroma volatiles are discussed below in detail.

#### 2.2.1. Esters

Esters are the characteristic volatile compounds that define strawberry volatiles, and they represent the most abundant volatile compounds in strawberries, with as many as 131 different types, comprising 25% to 90% of total strawberry fruit volatiles [25]. Esters are also the major source of fruity and floral odors in strawberries, and the content of esters may be the basis for classifying different volatile patterns [3,26,27]. The most important odorants of *F. × ananassa* are methyl butanoate, ethyl butanoate, ethyl hexanoate, methyl hexanoate, hexyl acetate, and 2-methylbutanoate [3,28]. In the present work, total esters comprised 0.35%, 17.48%, 19.0%, 24.05%, and 34.34 % of total volatiles in microwave-dried, oven-dried (60 °C), oven-dried (45 °C), freeze-dried, and shade air-dried strawberry samples, respectively, with respect to 36.96% in fresh samples. Shade air-drying was the best method of drying in terms of total ester content, which was only slightly decreased with respect to the control (fresh strawberry). The most abundant esters present in the fresh fruit were *trans*-2-hexenyl acetate (13.70%), hexyl acetate (12.68%), ethyl acetate (2.56%), ethyl hexanoate (2.17%), *cis*-2-hexenyl acetate (1.52%), octyl formate (1.51%), octyl acetate (0.99%), hexyl butanoate (0.48%), methyl butanoate (0.24%), methyl hexanoate (0.12%), and ethyl butanoate (0.11%); these finding are consistent with the literature [3,18,23]. These compounds have aroma properties with fruity notes, and most of them are among the most important flavor notes in terms of the aroma values [11]. The most abundant esters present in the microwave-dried, oven-dried (60 °C), oven-dried (45 °C), freeze-dried, and shade air-dried strawberry samples were (*E*)-2-hexenyl hexanoate (0.16%), ethyl hexanoate (8.91%), ethyl hexanoate (12.89%), *trans*-2-hexenyl acetate, and ethyl hexanoate (20.54%), respectively. Microwave-drying, oven-drying (60 °C), oven-drying (45 °C), and freeze-drying led to a significant reduction in fruity ester notes (105.60-, 2.24-, 1.76-, and 1.54-fold less, respectively) compared with the control, indicating microwave-drying as the worst method, which showed a drastic reduction in ester content (total esters; 0.35%). In contrast, the fruity compounds were well retained in the shade air-dried sample, followed by freeze-dried strawberry product. In addition, frozen strawberry sample (1 week freezing) showed a moderate reduction in ester content (21.38%) compared with the control (36.96%), and this is consistent with the literature [28]. It has been reported that concentrations of esters present in fresh strawberries are superior to those in frozen strawberries, and there was a decrease of 80% in ester content after 1 day of freezing [29,30]. In contrast to this, it was found that the levels of ethyl butanoate in frozen strawberries was higher than that in fresh strawberries [30,31], and these findings totally agree with our results. The reason for this phenomenon is the oxidation of acids following the thawing process of the frozen strawberries. It can be noted that the ethyl acetate content decreased in the microwave-dried, oven-dried (60 °C), oven-dried (45 °C), and freeze-dried samples compared to the fresh control, but air-dried samples showed an equal amount to the fresh fruits. Ethyl acetate was identified as an off-flavor note in strawberries [11].

#### 2.2.2. Furanones

Strawberries contain very low amounts of volatile furanones. However, compared to their threshold values, the furanones as a small group of aroma volatiles significantly contribute to the overall strawberry aroma [3,28]. 2,5-Dimethyl-4-hydroxy-3(2*H*)-furanone (furaneol, DMHF) and 2,5-dimethyl-4-methoxy-3(2*H*)-furanone (mesifurane, DMMF) are considered the two most important furanones in strawberry, and both of them characterized by a caramel-like and sweet aroma and flavor impression [3]. These compounds have a strong “strawberry-like” aroma with extremely low aroma threshold values; thus, they are also termed strawberry furanone (DMHF) and berry furanone (DMMF) [25,32]. Mesifurane is more stable than furaneol; although it resembles the typical aroma of wild strawberry, a much higher concentration of this compound has been found in some cultivated varieties [25]. In the current study, four furanones, namely, mesifurane, furaneol, 2,4-dihydroxy-2,5-dimethyl-3(2*H*)-furan-3-one, and dihydro-5-methyl-5-vinyl-2(3*H*)-furanone, were detected in the analyzed samples, accounting for 0.15%, 0.94%, 1.99%, 3.27%, 10.77%, and 11.44% of total volatiles in frozen, oven-dried (60 °C), microwave-dried, freeze-dried, shade air-dried, and oven-dried (45 °C) strawberry samples, respectively, compared to 0.10% in fresh samples. Mesifurane and furaneol were found in all samples, while 2,4-dihydroxy-2,5-dimethyl-3(2*H*)-furan-3-one was only detected in oven-dried (60 °C) and oven-dried (45 °C) strawberry samples, and dihydro-5-methyl-5-vinyl-2(3*H*)-furanone was only present in microwave- and shade air-dried samples. Mesifurane was the most abundant furanone, followed by furaneol. It has been reported that mesifurane levels increase during fruit ripening while furaneol simultaneously decreases [25], but both show similarly low odor threshold values [28,33]. The highest percentage of mesifurane was present in oven-dried (45 °C) (9.30%), followed by shade air-dried (8.47%) samples, and the lowest percentage was detected in fresh strawberry (0.08%). The highest percentage of furaneol was found in shade air-dried strawberry (1.92%), followed by oven-dried (45 °C) (1.89%), freeze-dried (0.04%), oven-dried (60 °C) (0.02%), frozen (0.02%), and fresh (0.02%) samples. Interestingly, furanone levels were increased in all drying methods especially heat-drying treatments at low (oven-drying, 45 °C) or room temperature (shade air-drying) and freeze-drying; consequently, the “strawberry-like” note or aroma would be increased or at least maintained and retained in these powders because it was reported that the abundance of furaneol and mesifurane strongly contributed to “fresh strawberry” sensory attributes [28,34]. Furaneol was detected in trace amounts in oven-dried (60 °C) and was not detected in microwave-dried samples due to the high microwave temperature, and this is consistent with other authors who reported that furaneol is not stable and its destruction depends on temperature and pH [25,35,36]. Furthermore, due to its sensitivity in aqueous solutions, furaneol detection is affected by the conditions of extraction or isolation and detection technique [25].

#### 2.2.3. Lactones

Lactones are cyclic esters and are structurally related to furanones; however, for the purpose of discussion, they were classified as a separate group. They contribute to fruity and coconut aromas. This chemical class is one of the most intense and important strawberry aroma volatiles. *γ*-Decalactone represents the major compound, and it can be used for variety classification [25,26,28,32,37]. It has been reported that the amounts of individual lactones vary according to type of cultivar; for example, Independence, Camarosa, and Hood strawberry cultivars were characterized by higher levels of lactones than other cultivars [25]. Moreover, Hood and Independence strawberry cultivars had higher total odor activity values (OAVs) for lactones, which correlated well with perceived high peach note levels in the sensory study [25]. In this work, six lactones (*γ*-butyrolactone, *γ*-caprolactone, *γ*-octalactone, *γ*-nonalactone, *γ*-decalactone, and *γ*-dodecalactone) were detected, representing 30.05%, 14.82%, 13.83%, 8.62%, 7.18%, and 4.80% of total volatiles in frozen, freeze-dried, shade air-dried, microwave-dried, oven-dried (45 °C), and oven-dried (60 °C) strawberry samples, respectively, with respect to 4.07% in fresh sample. *γ*-Decalactone was the major lactone in frozen (26.05%), freeze-dried (12.14%), shade air-dried (8.60%), and oven-dried (45 °C) (4.78%) samples, while *γ*-butyrolactone was the major lactone in microwave-dried (4.72%), oven-dried (60 °C) (3.15%), and fresh (3.11%) samples. *γ*-Decalactone possesses a “peach”, “strawberry-like” aroma and is among the key strawberry flavor volatiles in many cultivars [32]. Noteworthily, the levels of lactones were significantly increased in all treatments, particularly frozen, freeze-dried, and shade air-dried samples. 

#### 2.2.4. Terpenes

Terpenes are of particular interest in strawberries since these compounds have characteristic sensory properties and are known to exert antimicrobial activity [28]. Among these compounds, volatile monoterpenes (C10) and sesquiterpenes (C15) were identified in most soft fruits. The monoterpene linalol and the sesquiterpene nerolidol are the main and most important volatile terpenes in cultivated strawberry fruits, whereas *α*-pinene, *β*-myrcene, *α*-terpineol, *β*-phellandrene, and myrtenyl acetate have been identified in wild strawberries [3,20]. In the current study, a total of 33 terpenoid compounds were detected in all the analyzed strawberry samples, accounting for 41.57%, 39.89%, 23.32%, 19.48%, 16.14%, and 15.14% of total volatiles in frozen, freeze-dried, microwave-dried, oven-dried (60 °C), oven-dried (45 °C), and shade air-dried strawberry samples, respectively, with respect to 18.28% in fresh samples. It is noticeable that total terpene percentage increased after freezing, freeze-drying, microwave-drying, and oven-drying (60 °C), whereas oven-drying (45 °C), and shade air-drying decreased this percentage. As for the effect of temperature on volatile terpenes, a previous report revealed that the terpene content in the “Akihime” strawberry cultivar after 9 days of storage at low temperature declined less than that at room temperature [38], whereas another study found that, in white “Sweet Charlie” fruit cultivar, after storage at 15 and 25 °C, terpene level increased with elevation in temperature [3,39]. In this study, linalol was the major terpene in the fresh strawberry fruit followed by nerolidol, and it was reported that linalol is present in large amounts in some strawberry cultivars [32]. Interestingly, the intensity of nerolidol in all treatments was much greater than in the fresh strawberry; while linalol intensity decreased in all samples except in the freeze-dried sample. In a previous study, terpenes were reported as the least intense aroma components contributing to floral aroma notes of strawberries [32]. Linalol was classified as a citrus, floral [32], or lemon-like aroma note [11], and nerolidol was categorized as a fir/pine-like aroma [11]. In addition to the reasonably high amounts of the monoterpene linalol and the sesquiterpene nerolidol, these two compounds were accompanied by small or trace amounts of other terpenes, such as *α*-terpineol, d-limonene, caryophyllene, *cis*-*β*-farnesene, myrtenol, myrtenyl acetate, and terpinolene, and these results are consistent with the literature [26,28]. *α*-Terpineol has been reported as a potential off-flavor metabolite in strawberry juice [28,34]. In general, esters were the major class and were found in high contents in fresh strawberry fruit (36.96% of total volatile fraction); however, after freeze-drying and freezing (1 week), terpenes became the major class, corresponding to 39.89% and 41.57% of the total volatile fraction, respectively (Table 1). Relatively high concentrations of terpenes could be used to distinguish among genotypes such as Senga Sengana, Bounty, and Jonsok, whereas other compounds related to the class of esters and acids might also be used for classification purposes [28,40]. 

#### 2.2.5. Aldehydes and Ketones

In this study, 19 aldehydes (for example, hexanal, (*E*)-2-hexenal, benzaldehyde, 3,4-dimethyl benzaldehyde, nonanal, (*E*)-2-nonenal, decanal, furfural, and 5-hydroxymethylfurfural) were identified in all treatments, covering 0.74%, 4.16%, 6.81%, 17.05%, 24.10%, and 29.09% of total volatiles in frozen, shade air-dried, freeze-dried, oven-dried (45 °C), oven-dried (60 °C), and microwave-dried samples, respectively, compared to 0.82% in fresh samples. An increase in aldehyde content was observed during all drying treatments, particularly at elevated temperatures (oven-dried (45 °C), oven-dried (60 °C), and microwave-dried samples), while aldehyde content decreased in only the frozen sample. Hexanal and (*E*)-2-hexenal are primarily responsible for the green/vegetative aroma component in strawberries. (*E*)-2-Hexenal is thought to be responsible for the “fresh strawberry” flavor in “Strawberry Festival” and “Florida Radiance” strawberries [32]. It is also worth noting that some aldehydes such as furfural and 5-hydroxymethylfurfural appeared only in heat-drying methods such as oven- and microwave-drying, and these results are in agreement with other authors who reported that 5-hydroxymethylfurfural (HMF) and furfural are heat-induced chemical markers generated during strawberry jam processing and baking of cereal-based products [16,41].

For ketones, the heat-drying methods showed a significant increase in ketone content, but freezing and freeze-drying decreased the ketone content. Microwave-dried samples (14.57%) possessed the highest total ketone content, whereas frozen (0.02%) and freeze-dried (0.43%) samples showed the lowest values compared to the fresh sample (0.78%). This demonstrated that drying at elevated temperatures altered the overall flavor impression in dried samples by enriching ketone and aldehyde flavor notes, and this is consistent with the literature [11].

#### 2.2.6. Alcohols and Acids

Alcohols, together with esters, furanones, and aldehydes, are the compounds that mainly determine strawberry aroma. Alcohols account for as much as 35% of the volatiles, but normally contribute little to strawberry aroma [42]. In this study, 16 alcohols were detected in all the analyzed samples; 1-hexanol, 1-octen-3-ol, 1-heptanol, (*Z*)-3-hexen-1-ol, (*E*)-2-hexen-1-ol, 1-octanol, and phenyl ethyl alcohol were the major ones. A high alcohol content was maintained in the fresh sample (17.10%), but showed a decrease in percentage in all treatments as follows: microwave (8.24%), natural air-dried (5.91%), oven-dried (60 °C) (3.46%), oven-dried (45 °C) (2.18%), freeze-dried (1.15%), and frozen (0.09%) samples. The major alcohol in fresh strawberry was 1-hexanol (14.37%) which displayed a drastic reduction in content in natural air-dried (1.14%), freeze-dried (0.35%), and frozen (0.03%) samples; it was not detected in artificial heat-drying methods (oven and microwave).

The unsaturated alcohol, (*Z*)-3-hexenol, is an important contributor to the green, leafy aroma in strawberry [32]. This compound was detected in fresh strawberry, as well as frozen and freeze-dried samples, while it was undetectable in the other heat-dried samples, and this is consistent with the literature [43].

With respect to acids, all the current treatments increased the acid content compared to fresh sample (0.12%); oven-drying (45 °C) (8.79%) showed the highest acid content, and microwave-dried sample demonstrated the lowest value (0.13%). Different aromas are given by volatile organic acids, but they are minor components of strawberry aroma [32]. The level of hexanoic acid (off-flavor), which contributes to an unpleasant aroma impression [33,43], was increased in all samples, with the highest value in oven-drying (60 °C) (0.89%).

### 2.3. Application of the HS-SPME/GC-MS Method to Strawberry Jams

Strawberry is a very delicious fruit, cultivated in nearly all countries of the world. Strawberry has always been a favored raw material for the production of various food products such as jams and juice, due to its unique aroma and attractive color. Aroma is a prerequisite for quality control; thus, its stability and retention in various food products is of great interest due to its strong relationship with consumer’s acceptability of foods. During preparation of fruit products, different additives are used that influence food product physicochemical properties, aroma, texture, and color [13]. It is generally known that the aroma and flavor of fruit products changes drastically during manufacturing, processing, and storage. Various compounds have been reported to degrade over time, whereas others are formed. In most cases, these degradation and formation reactions lead to a deterioration of the original aroma of the product. Consequently, the shelf-life of commercially available fruit jams, juices, and nectars is not limited by microbial spoilage but by a drastic decrease in the sensory quality of the products due to chemical reactions [44].

In the present work, the nature of the aroma present in strawberry foodstuff such as jam was investigated. A summary of aroma active volatiles from the various strawberry jams analyzed by the SPME/GC-MS technique is shown in Table 1. According to the relative percentage of volatiles present in the analyzed strawberry jams, terpenoids were the most abundant class in Jam 2 and Jam 1, accounting for 79.24% and 53.31% of the total volatile composition, respectively, and these results are consistent with a previous report where terpenes were found as the most dominant class in jams of cranberry, blueberry, raspberry, and blackberry/blackcurrant [45]. On the other hand, acids and aldehydes were the most abundant classes in Jam 3 (32.03%) and Jam 4 (27.86%), respectively, followed by terpenes which accounted for 30.31% and 26.86% of the total volatile fraction, respectively. Nerolidol was the most dominant terpene in Jam 1 (40.99%), Jam 4 (17.92%), and Jam 2 (13.75%), while limonene was the highest terpene in Jam 2 (58.05%). Linalol was detected in a moderate amount in jam 3 (5.18%), and in low levels in Jam 1 (0.70%) and Jam 4 (0.49%), while it was not detected in Jam 2. Terpenes, such as linalol and nerolidol, contributed with floral, and citrus aroma notes. It is worth noting that *α*-terpineol was detected in high amounts in all analyzed strawberry jams in a range from 2.09% to 8.75%. *α*-Terpineol has been reported as a potential off-flavor metabolite in strawberry juice [28,34], and it contributes with a “woody” and “piney” aroma note [45]. 

Aldehydes such as furfural, benzaldehyde, and 5-hydroxymethylfurfural were detected in all jam samples. Furfural showed the highest amount, followed by benzaldehyde and 5-hydroxymethylfurfural, and Jam 4 was the richest one in these three aldehydes with concentrations of 17.22%, 9.8%, and 0.5%, respectively. Thermal treatment might further lead to the formation of new volatile structures; for example, 5-hydroxymethylfurfural (HMF) and furfural are heat-induced chemical markers generated during strawberry jam preparation and baking of cereal-based products [16,17,28,41]. Therefore, the high levels of furfural and 5-hydroxymethylfurfural in jams are justified due to heat treatment under jam processing. Similarly, these two compounds were detected only in heat-drying methods such as oven and microwave and not detected in freeze-dried and frozen samples.

Esters, imparting important fruity attributes to foods, were not detected in all jam samples except Jam 2 which possessed only traces (0.12%), compared to 36.96% in fresh strawberry fruits. Furthermore, alcohols were totally absent in Jams 3 and 4, while they were found in small amounts or traces in Jam 1 (0.25%) and Jam 4 (0.09%) compared to fresh fruits (17.10%). This means that esters and alcohols in most jam samples were destroyed during strawberry jam processing, and these compositional changes in jam would be responsible for a poorer sensory impression.

Additionally, furanones were not detected in all jams except Jam 3, which showed a higher furanone content represented by mesifurane (5.34%) than the fresh fruits (0.10%). In contrast, acid content increased in all jam samples, whereby Jam 3 showed the highest acid amount (32.03%), and Jam 2 demonstrated the lowest acids level (3.48%), compared to 0.12% in fresh fruits. The acids detected were hexanoic acid, acetic acid, octanoic acid, and 2-methylbutanoic acid, with hexanoic acid being the major one in Jam 4 and Jam 3, accounting for 17.59% and 19.96%, respectively. Hexanoic acid was reported as an off-flavor which contributes to an unpleasant aroma impression [33,43]. 

Lactones, including *γ*-butyrolactone, *γ*-caprolactone, *γ*-decalactone, and *γ*-dodecalactone were identified in the jam samples (Table 1). Total lactone amounts were increased in all jam samples except Jam 2, compared to fresh fruits. It was reported previously that lactones were correlated with peach note levels in the sensory study [25].

Lastly, strawberry aroma was modified by heat treatment during jam processing. In fact, the volatile compounds characteristic of fresh strawberries were mostly replaced in processed strawberry by certain heat-induced volatile compounds, such as furfural and 5-hydroxymethylfurfural, by the disappearance of other compounds (e.g., esters were missed except traces in Jam 2, as well as most alcohols), or by an increase (e.g., *γ*-decalactone, *γ*-dodecalactone, *D*-limonene, (*E*)-nerolidol, *α*-terpineol, hexanoic acid) or decrease (e.g., *γ*-butyrolactone, linalol, 1-hexanol) in some existing compounds, which led to a poorer sensory impression compared to fresh fruits; these results are in a good accordance with other authors [16,17,28]. These compounds (e.g., furfural and 5-hydroxymethylfurfural) are a key source of flavor and color and are responsible for the enjoyment of most heat-processed foods, but some of them are also regarded as carcinogenic in such a way that their control is necessary [17]. Interestingly, natural strawberry aroma compounds are mostly replaced by Maillard reaction products, most of them with carcinogenic properties, in commercial jam. For these reasons, shorter heating periods using whatever the temperature applied represent an alternative to industrial jam processing to obtain a healthier and more pleasant product; in this case, homemade jam using low temperature is more favored as the natural strawberry aroma compounds will be more preserved and retained.

### 2.4. Principal Component Analysis (PCA)

**Figure 3:**
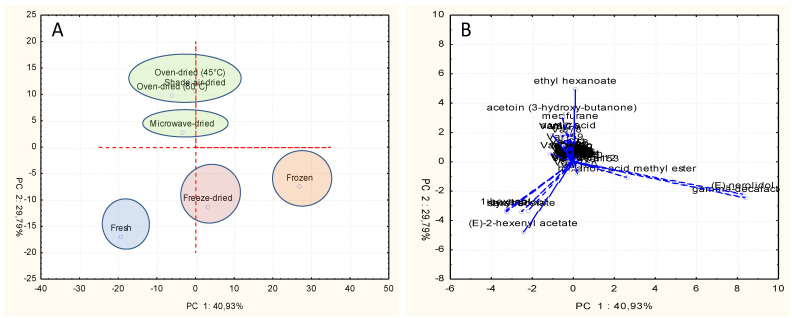
(**A**) Score plot representing the distribution of strawberry samples. (**B**) Loading plot for the correlation between variables of the matrix.

PCA allowed us to differentiate between the different treatments of strawberry according to their volatile constituents. The PCA score and loading plots, including relationships between strawberry samples and volatile components, are reported in Figure 3. The sum of the first two principal components (PCs) explained 70.72% of data variability. Graphs showed that the fruit pretreatment had a significant effect on the volatile profile of strawberry, resulting in four different groups. The variability of data was produced mostly by variance of (*E*)-nerolidol (values of eigenvectors: 8.27; −2.21) and *γ*-decalactone (8.39; −2.45) in the first PC, and by ethyl hexanoate (0.10; 4.91) and (*E*)-2-hexenyl acetate (−2.43; −4.78) in the second PC. The group located in the upper side of the score plot was composed of shade air-dried or oven-dried (45 and 60 °C) samples. They were mostly correlated with a high release of ethyl hexanoate, acetoin (3-hydroxy-butanone), and mesifurane. Another group present in the middle upper side of the score plot was composed of microwave-dried samples and characterized by mesifurane. On the other hand, fresh, freeze-dried, and frozen samples grouped separately in the lower side of the score plot, with the former being characterized by (*E*)-2-hexenyl acetate, 1-hexanol, ethyl hexanoate, and hexyl acetate, the latter being characterized by (*E*)-nerolidol and *γ*-decalactone, and the freeze-dried samples being characterized by intermediate composition.

According to PCA, we can conclude that the retention of fruit volatiles in dried strawberry fruits varied according to the type of drying method and freezing. A previous study classified the strawberry aroma active volatiles into six aroma groups according to their primary aroma characteristics as follows: fruity (comprising more than 50% of the total aroma intensity, represented by esters and lactones), sweet (about 18%, covered by furanones), floral (the least intense strawberry aroma class, 7%, featuring terpenes), volatile acids (9%), green (aldehydes, 10%), and miscellaneous [32]. Accordingly, the PCA (Figure 3) and obtained results (Table 1), concerning the contents of esters, lactones, and furanones, showed that shade air-dried, followed by frozen, freeze-dried, and oven-dried 45 °C samples, retained more of the fruity and sweet aromas of strawberry fruits, representing more than 68% of the total aroma intensity according to the literature [32]. In contrast, the microwave-drying method showed a drastic loss of fruity esters volatiles, while the oven-dried 60 °C sample demonstrated an intermediate loss.

## 3. Materials and Methods 

### 3.1. Chemicals

The alkane mixture was purchased from Sigma-Aldrich (Milano, Italy). The SPME fibers used (Supelco, Bellofonte, PA, USA) were the following: divinylbenzene/carboxen/polydimethylsiloxane (DVB/CWR/PDS) 80 μm and polydimethylsiloxane/divinylbenzene (PDMS/DVB) 65 μm.

### 3.2. Strawberry Samples 

**Figure 4:**
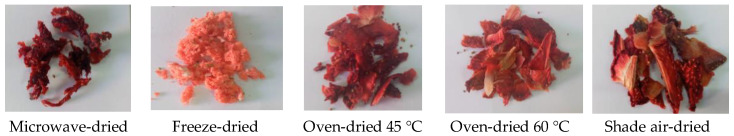
Appearance of dried strawberry fruits treated by different drying methods.

Fresh ripe fruits of strawberry (*Fragaria* × *ananassa*) were purchased from the supermarkets of Camerino (Marche, Italy). Whole fruits of uniform size, shape, and ripening stage were selected, washed, and manually fractioned vertically into small pieces (3-mm thick) using a knife. Then, 100 g of freshly cut fruits were stored in a freezer at −18 °C for 1 week (frozen sample). Freshly cut fruits pieces (100 g) were dried to constant weight artificially using a BUCHI Lyovapor™ L-200 freeze-dryer (Büchi Labortechnik AG, Flawil, Switzerland) at −54 °C, with a shelf temperature of 10 °C and a pressure of 0.05 mbar for 48 h, microwave oven (Delonghi MWJ63, 900 W, De’Longhi S.P.A, Treviso, Italy) for 4 min, an electric oven (Model: FD 56, Binder, Germany) at 45 °C and 60 °C for 24 h and 16 h, respectively, or naturally using the shade air-drying method for 96 h, where cut pieces were spread on watch glasses and then dried indoors to allow the moisture to evaporate naturally [46]. Then, dried fruit pieces (Figure 4) were ground using a mortar and pestle to give a powder. Dried powders were sealed and stored at 4 °C until analysis. The suitability of the HS-SPME extraction and GC methods was evaluated by analyzing a number of strawberry jams purchased from different commercial local markets of Camerino. The ingredients listed on the labels of the jams are shown in Table 2. Commercial jam samples were stored at 4 °C prior to analysis.

### 3.3. Headspace Solid-Phase Microextraction (HS-SPME)

The fresh, frozen, and dried powdered fruits of the plant were used for HS-SPME coupled with GC-MS. The sample (5 g fresh or frozen, or 2.5 g dried) was placed in a 20 mL vial which was tightly capped with a PTFE/silicon septum. The analysis was performed using a PAL RTC 120 autosampler (CTC Analytics AG, Zwingen, Switzerland), which was able to yield strong analysis repeatability. The influence of the extraction temperature on the adsorption of the volatile compounds was first evaluated, and the extraction time was fixed at 30 min, in addition to equilibrium time of another 20 min before the extraction process. The incubation of the sample was performed at different temperatures (60 and 80 °C) under agitation (250 rpm, 5 s of on-time and 2 s of off-time), and extracted using two SPME fiber coatings (DVB/ CWR/PDS 80 μm, and PDMS/DVB 65 μm). Then, different extraction times (15 and 30 min) were examined at 60 °C (the optimum extraction temperature obtained from the previous analysis) using DVB/CWR/PDMS 80 μm (the optimum SPME fiber obtained from the previous analysis). Lastly, the effect of NaCl addition was evaluated using the optimal extraction temperature (60 °C), fiber (DVB/CWR/PDMS 80 μm), and time (30 min) obtained previously. The fibers were conditioned for 20 min at 250 °C and then inserted inside the headspace of sample vial with a speed of 20 mm·s^−1^ and a penetration depth of 40 mm. The extraction was performed and then the fiber was inserted into the injector port at a speed of 100 mm·s^−1^ and a penetration depth of 40 mm. The desorption occurred at 250 °C for 3 min. After desorption, the fiber was conditioned at 250 °C for 15 min.

### 3.4. GC-MS Analysis

The GC-MS analysis was carried out using an 8890 gas chromatograph (GC) from Agilent equipped with a PAL RTC 120 autosampler and a 5977B mass spectrometer (MSD) Agilent (Santa Clara, CA, USA). The ionization was obtained by using an electron ionization source (EI). The injector temperature was set at 250 °C, and the liner used was recommended for SPME injection, namely, Inlet liner, Ultra Inert, splitless, straight, 0.75 mm id, from Agilent. The gas carrier was helium at flow rate of 1 mL·min^−1^. The separation of target molecules was established onto an HP-5 capillary column (30 m, 250 µm i.d., 0.25 µm film thickness). The oven temperature program started at 35 °C for 3 min, before increasing from 35 to 140 °C at 3 °C/min and from 140 to 300 °C at 15 °C/min, followed by a hold at 300 °C for 5 min. The temperatures of the ionization source and the mass analyzer were set at 230 and 150 °C, respectively. The acquisition was carried out in SCAN mode (35–450 *m*/*z*). The peak or compound identification was performed by comparison of the mass spectra and experimental retention indices (RI) with data of the NIST library (US National Institute of Standards and Technology) and those reported in the literature. The relative percentages of the individual components were calculated on the basis of GC peak area, which was obtained by dividing the area of each component by the total area of all separated components. Percentage values were the means of two replicates for each sample. Data results were managed using MSD ChemStation Software (Agilent, Version G1701DA D.01.00, Santa Clara, CA, USA).

### 3.5. Principal Component Analysis (PCA)

In order to find possible correlations between samples depending on diverse pretreatments, the volatile compositions were analyzed by PCA using a covariance matrix including 161 variables × 7 samples (1127 data). For this purpose, the software Statistica v. 7.1. (Stat Soft Italia, Vigonza, Italy) was used to generate two-dimensional score and loading plots. Eigenvalues were calculated using a covariance matrix among 161 chemical compounds as input, and the two-dimensional PCA biplot, including both samples of different drying and freezing treatments and compounds, was generated. Jam samples and the four compounds detected only in jams were excluded from PCA analysis, because jams did not undergo our drying or freezing treatments.

## 4. Conclusions

In the present study, HS-SPME/GC-MS and principal component analysis were used to investigate the influence of freezing and different drying methods (oven-drying at 45 °C and 60 °C, freeze-drying, microwave-drying, shade air-drying) for the production of strawberry powders with desirable aroma, which is one of the most important quality attributes. In addition, the volatile profiles of some commercial strawberry jams were analyzed. A total of 165 compounds were identified, accounting for 85.03–96.88% of the total headspace compositions. Our study showed that freezing and different drying methods exerted different influences on types and the content of volatile compounds of strawberry, and distinct separations between the different strawberry treatments were generated. The results and PCA revealed that the most remarkable representative differential volatiles were ethyl hexanoate, hexyl acetate, (*E*)-2-hexenyl acetate, mesifurane, (*E*)-nerolidol, *γ*-decalactone, 1-hexanol, and acetoin. The PCA and obtained results showed that shade air-dried, frozen, freeze-dried, and oven-dried 45 °C samples retained more of the fruity and sweet aromas of strawberry fruits, representing more than 68% of the total aroma intensity according to the literature. In contrast, the microwave-drying method showed a drastic loss of fruity esters volatiles, while the oven-dried 60 °C sample demonstrated intermediate loss. Strawberry jams demonstrated almost a complete destruction of esters and alcohols in most jam samples, while terpenes were significantly increased; in addition, off-flavor and heat-induced volatiles, such as furfural and 5-hydroxymethylfurfural, were generated. These findings help better understand the aroma of strawberry and provide a guide for the effects of drying, freezing, and jam processing. Each drying process may be more suitable for the production of a specific targeted flavor food ingredient, as a distinct volatile composition was observed in each strawberry powder. The produced powders could be added to different food products such as baking and dairy products, to improve their functional value by adding desirable strawberry flavor.

## Figures and Tables

**Table 1:** Headspace volatile organic components identified in fresh, frozen and dried strawberry and their relative percentages of area (%); relative standard deviation percentages (RSDs%, *n* = 2) were ranged from 0.83 to 9.31.

No.	Compound Name and Class	RI Calc.	Fresh	Frozen	Freeze-Dried	Microwave-Dried	Oven-Dried (60 °C)	Oven-Dried (45 °C)	Shade Air-Dried	Jam 1	Jam 2	Jam 3	Jam 4
	**Furanones**		**0.10**	**0.15**	**3.27**	**1.99**	**0.94**	**11.44**	**10.77**	**0.00**	**0.00**	**5.34**	**0.00**
1	2,4-Dihydroxy-2,5-dimethyl-3(2*H*)-furan-3-one	976	0.00	0.00	0.00	0.00	0.11	0.25	0.00	0.00	0.00	0.00	0.00
2	Dihydro-5-methyl-5-vinyl-2(3*H*)-furanone	1037	0.00	0.00	0.00	0.41	0.00	0.00	0.38	0.00	0.00	0.00	0.00
3	Mesifurane	1058	0.08	0.13	3.23	1.58	0.81	9.30	8.47	0.00	0.00	5.34	0.00
4	Furaneol	1066	0.02	0.02	0.04	0.00	0.02	1.89	1.92	0.00	0.00	0.00	0.00
	**Lactones**		**4.07**	**30.05**	**14.82**	**8.62**	**4.80**	**7.18**	**13.83**	**18.12**	**1.32**	**22.11**	**11.81**
5	*γ*-Butyrolactone	923	3.11	0.00	1.85	4.72	3.15	1.67	4.33	0.67	0.19	0.00	3.48
6	*γ*-Caprolactone	1050	0.00	0.00	0.09	0.09	0.21	0.00	0.16	0.00	0.00	0.00	0.05
7	*δ*-caprolactone	1088	0.00	0.00	0.14	0.42	0.09	0.34	0.07	0.00	0.00	0.00	0.00
8	*γ*-Octalactone	1250	0.02	0.00	0.11	0.00	0.00	0.00	0.00	0.00	0.00	0.00	0.00
9	*δ*-Octalactone	1276	0.00	0.00	0.00	0.00	0.01	0.04	0.07	0.00	0.00	0.00	0.00
10	*γ*-Nonalactone	1353	0.01	0.00	0.00	0.00	0.00	0.00	0.06	0.00	0.00	0.00	0.00
11	*γ*-Decalactone	1459	0.89	26.08	12.14	3.15	1.28	4.78	8.60	15.22	0.35	20.76	7.47
12	*γ*-Dodecalactone	1677	0.04	3.97	0.49	0.24	0.06	0.35	0.54	2.23	0.78	1.35	0.81
	**Esters**		**36.96**	**21.38**	**24.05**	**0.35**	**17.48**	**19.00**	**34.34**	**0.00**	**0.10**	**0.00**	**0.00**
13	Ethyl acetate	610	2.56	0.46	0.29	0.00	0.00	0.00	2.50	0.00	0.00	0.00	0.00
14	Methyl butanoate	721	0.24	0.46	0.09	0.00	0.00	0.00	0.00	0.00	0.00	0.00	0.00
15	Methyl 2-methylbutanoate	777	0.00	0.16	0.00	0.00	0.00	0.00	0.00	0.00	0.00	0.00	0.00
16	Ethyl butanoate	804	0.11	0.99	0.04	0.00	0.27	0.06	1.10	0.00	0.00	0.00	0.00
17	Butyl acetate	816	0.02	0.07	0.00	0.00	0.00	0.00	0.00	0.00	0.00	0.00	0.00
18	Isobutyl butyrate	843	0.01	0.13	0.00	0.00	0.00	0.00	0.00	0.00	0.00	0.00	0.00
19	Ethyl 2-methylbutanoate	850	0.00	0.33	0.00	0.00	0.00	0.00	0.51	0.00	0.00	0.00	0.00
20	Methyl (*S)-(+*)-3-hydroxybutyrate	853	0.01	0.00	0.00	0.00	0.00	1.24	0.00	0.00	0.00	0.00	0.00
21	2-Methylbutyl acetate	880	0.01	0.04	0.05	0.00	0.00	0.00	0.00	0.00	0.00	0.00	0.00
22	Methyl hexanoate	925	0.12	8.00	1.95	0.00	0.25	0.37	0.19	0.00	0.00	0.00	0.00
23	Ethyl 3-hydroxybutyrate	936	0.00	0.00	0.00	0.00	0.27	0.00	0.12	0.00	0.00	0.00	0.00
24	Ethyl hexanoate	1000	2.17	4.65	0.79	0.11	9.91	10.89	18.54	0.00	0.00	0.00	0.00
25	Hexyl acetate	1014	12.68	0.82	4.66	0.00	0.63	0.76	1.52	0.00	0.00	0.00	0.00
26	(*E*)-2-Hexenyl acetate	1016	13.70	1.15	10.76	0.00	0.73	0.75	0.50	0.00	0.00	0.00	0.00
27	(*Z*)-2-Hexenyl acetate	1020	1.52	0.00	1.82	0.00	0.00	0.00	0.03	0.00	0.00	0.00	0.00
28	Isopropyl hexanoate	1037	0.01	0.48	0.33	0.00	0.00	0.00	0.00	0.00	0.00	0.00	0.00
29	Ethyl 2-hexenoate	1043	0.01	0.00	0.00	0.00	0.00	0.00	0.06	0.00	0.00	0.00	0.00
30	Formic acid octyl ester	1069	1.51	0.00	0.00	0.00	0.00	0.00	0.00	0.00	0.00	0.00	0.00
31	Propyl hexanoate	1093	0.01	0.00	0.00	0.00	0.00	0.00	0.13	0.00	0.00	0.00	0.00
32	Acetic acid heptyl ester	1111	0.11	0.00	0.04	0.00	0.00	0.00	0.00	0.00	0.00	0.00	0.00
33	Methyl octanoate	1122	0.02	1.35	0.59	0.00	0.05	0.55	0.00	0.00	0.00	0.00	0.00
34	Ethyl 3-hydroxyhexanoate	1124	0.01	0.00	0.00	0.00	0.00	0.00	0.15	0.00	0.00	0.00	0.00
35	Hexanoic acid-2- methyl propyl ester	1148	0.00	0.00	0.00	0.00	0.00	0.00	0.29	0.00	0.00	0.00	0.00
36	Benzoic acid ethyl ester	1165	0.06	0.02	0.05	0.00	0.29	0.23	0.33	0.00	0.00	0.00	0.00
37	Methyl salicylate	1187	0.02	0.00	0.18	0.00	0.00	0.00	0.00	0.00	0.00	0.00	0.00
38	Butanoic acid hexyl ester	1188	0.48	0.02	0.00	0.00	0.51	0.12	0.68	0.00	0.00	0.00	0.00
39	(*E*)-Butanoic acid-2-hexenyl ester	1192	0.11	0.00	0.00	0.00	0.19	0.04	0.03	0.00	0.00	0.00	0.00
40	Ethyl octanoate	1193	0.01	0.59	0.28	0.00	0.54	1.16	1.76	0.00	0.00	0.00	0.00
41	Octyl acetate	1207	0.99	0.65	0.69	0.00	0.00	0.00	0.00	0.00	0.00	0.00	0.00
42	1,4 butanediol diacetate	1212	0.00	0.00	0.13	0.00	0.00	0.00	0.00	0.00	0.00	0.00	0.00
43	Isopropyl octanoate	1229	0.01	0.04	0.00	0.00	0.00	0.00	0.00	0.00	0.00	0.00	0.00
44	Butanoic acid-3-methylhexyl ester	1237	0.05	0.00	0.00	0.00	0.00	0.00	0.07	0.00	0.00	0.00	0.00
45	Isopentyl hexanoate	1245	0.01	0.00	0.00	0.00	0.95	0.25	0.54	0.00	0.00	0.00	0.00
46	Pentyl hexanoate	1282	0.00	0.00	0.00	0.00	0.00	0.00	0.10	0.00	0.00	0.00	0.00
47	(*Z*)-3-Nonenyl acetate	1288	0.07	0.00	0.00	0.00	0.00	0.00	0.00	0.00	0.00	0.00	0.00
48	Ethyl nonanoate	1290	0.00	0.00	0.00	0.00	0.00	0.00	0.17	0.00	0.00	0.00	0.00
49	*n*-Nonyl acetate	1304	0.14	0.08	0.31	0.00	0.35	0.38	0.12	0.00	0.00	0.00	0.00
50	Methyl decanoate	1317	0.01	0.31	0.20	0.00	0.00	0.00	0.00	0.00	0.00	0.00	0.00
51	1,3 Pentanediol 2,2,4 trimethyl 1 isobutyrate	1364	0.00	0.00	0.08	0.00	0.00	0.16	0.00	0.00	0.00	0.00	0.00
52	*cis*-Methyl cinnamate	1373	0.01	0.00	0.31	0.00	0.23	0.11	0.04	0.00	0.00	0.00	0.00
53	*trans*-Methyl cinnamate	1373	0.01	0.04	0.00	0.00	0.00	0.00	0.00	0.00	0.00	0.00	0.00
54	Hexyl hexanoate	1379	0.04	0.05	0.28	0.08	0.74	0.33	0.55	0.00	0.00	0.00	0.00
55	(*2E*)-2-Hexenyl hexanoate	1381	0.05	0.06	0.03	0.16	0.38	0.17	0.00	0.00	0.00	0.00	0.00
56	Octyl butyrate	1382	0.02	0.00	0.00	0.00	0.00	0.00	0.36	0.00	0.00	0.00	0.00
57	Ethyl decanoate	1388	0.00	0.14	0.05	0.00	0.22	0.33	0.32	0.00	0.00	0.00	0.00
58	Ethyl cinnamate	1457	0.01	0.13	0.00	0.00	0.97	1.10	2.63	0.00	0.00	0.00	0.00
59	Propyl decanoate	1522	0.01	0.09	0.00	0.00	0.00	0.00	0.00	0.00	0.00	0.00	0.00
60	Octyl hexanoate	1581	0.01	0.07	0.00	0.00	0.00	0.00	0.00	0.00	0.10	0.00	0.00
61	Methyl tetradecanoate	1722	0.00	0.00	0.05	0.00	0.00	0.00	0.00	0.00	0.00	0.00	0.00
62	Ethyl-9-tetradecanoate	1771	0.00	0.00	0.00	0.00	0.00	0.00	0.12	0.00	0.00	0.00	0.00
63	Ethyl tetradecanoate	1792	0.01	0.00	0.00	0.00	0.00	0.00	0.75	0.00	0.00	0.00	0.00
64	Ethyl hexadecanoate	1985	0.00	0.00	0.00	0.00	0.00	0.00	0.13	0.00	0.00	0.00	0.00
	**Aldehydes**		**0.82**	**0.74**	**6.81**	**29.09**	**24.10**	**17.05**	**4.16**	**0.94**	**0.30**	**2.10**	**27.86**
65	3-Methyl butanal	656	0.00	0.00	0.07	0.22	0.28	0.27	0.00	0.00	0.00	0.00	0.00
66	Hexanal	801	0.14	0.11	0.15	1.25	0.32	0.50	0.20	0.00	0.00	0.00	0.00
67	Furfural	820	0.00	0.00	0.00	3.41	11.75	0.44	0.00	0.59	0.21	0.68	17.22
68	(*E*)-2-Hexenal	851	0.24	0.00	0.00	0.00	0.00	0.19	0.00	0.00	0.00	0.00	0.00
69	Heptanal	902	0.00	0.00	0.12	0.11	0.22	0.17	0.03	0.00	0.00	0.00	0.00
70	(*Z*)-2-Heptenal	955	0.00	0.00	0.00	0.00	0.41	0.00	0.00	0.00	0.00	0.00	0.00
71	Benzaldehyde	955	0.20	0.30	3.94	10.80	0.00	0.47	0.86	0.33	0.08	1.15	9.68
72	Octanal	1002	0.00	0.00	0.00	1.26	0.00	0.00	0.00	0.00	0.00	0.00	0.00
73	Benzeneacetaldehyde	1039	0.00	0.00	0.00	0.00	0.06	0.10	0.00	0.00	0.00	0.00	0.09
74	(*E*)-2-Octenal	1055	0.00	0.06	0.00	0.00	0.77	0.18	0.00	0.00	0.00	0.00	0.00
75	Nonanal	1101	0.18	0.17	1.83	4.27	4.37	6.48	1.76	0.00	0.00	0.25	0.37
76	(*2E,4E*)-2,4-Octadienal	1104	0.00	0.00	0.00	0.46	0.00	0.00	0.00	0.00	0.00	0.00	0.00
77	(*E*)-2-Nonenal	1155	0.05	0.01	0.05	0.45	0.66	1.27	0.25	0.00	0.00	0.00	0.00
78	Decanal	1200	0.01	0.09	0.60	1.74	3.81	5.54	0.92	0.00	0.00	0.00	0.00
79	3,4-Dimethyl benzaldehyde	1205	0.00	0.00	0.00	3.96	1.02	0.98	0.00	0.00	0.00	0.00	0.00
80	5-Hydroxymethylfurfural	1220	0.00	0.00	0.00	0.06	0.15	0.04	0.00	0.02	0.01	0.02	0.50
81	(*E*)-2-Decenal	1255	0.00	0.00	0.00	0.96	0.16	0.06	0.11	0.00	0.00	0.00	0.00
82	Undecanal	1299	0.00	0.00	0.05	0.00	0.06	0.35	0.00	0.00	0.00	0.00	0.00
83	2-Undecenal	1355	0.00	0.00	0.00	0.14	0.06	0.01	0.03	0.00	0.00	0.00	0.00
	**Ketones**		**0.78**	**0.02**	**0.43**	**14.57**	**5.97**	**10.54**	**5.14**	**0.87**	**0.28**	**3.03**	**3.45**
84	3-Pentanone	701	0.03	0.00	0.00	0.00	0.00	0.00	0.00	0.00	0.00	0.00	0.00
85	Acetoin	715	0.00	0.00	0.00	0.00	5.43	9.25	4.85	0.00	0.00	0.00	0.00
86	2-Methyl-1-penten-3-one	767	0.00	0.00	0.00	1.29	0.00	0.00	0.00	0.00	0.00	0.00	0.00
87	2-Heptanone	891	0.00	0.00	0.00	0.00	0.00	0.00	0.00	0.00	0.00	1.80	0.00
88	2-Acetoxy-3-butanone	896	0.00	0.00	0.00	0.00	0.07	0.09	0.09	0.00	0.00	0.00	0.00
89	4-Methyl-2-Heptanone	938	0.00	0.00	0.00	0.13	0.00	0.00	0.00	0.00	0.00	0.00	0.00
90	1-Octen-3-one	977	0.00	0.00	0.04	6.42	0.20	0.32	0.00	0.00	0.00	0.00	0.00
91	2-Methyl-3-octanone	984	0.00	0.00	0.06	0.91	0.00	0.00	0.00	0.00	0.00	0.00	0.00
92	6-Methyl-5-hepten-2-one	986	0.16	0.02	0.26	4.97	0.27	0.88	0.20	0.00	0.00	0.00	0.00
93	2*H*-Pyran-2,6(3*H*)-dione	998	0.00	0.00	0.00	0.00	0.00	0.00	0.00	0.87	0.28	0.00	0.00
94	Acetophenone	1060	0.59	0.00	0.00	0.55	0.00	0.00	0.00	0.00	0.00	0.00	1.64
95	2-Furyl hydroxy methyl ketone	1077	0.00	0.00	0.00	0.00	0.00	0.00	0.00	0.00	0.00	0.00	1.81
96	2-Nonanone	1090	0.00	0.00	0.00	0.00	0.00	0.00	0.00	0.00	0.00	1.23	0.00
97	6-Methyl-3,5-heptadiene-2-one	1110	0.00	0.00	0.00	0.16	0.00	0.00	0.00	0.00	0.00	0.00	0.00
98	Ketoisophorone	1138	0.00	0.00	0.07	0.14	0.00	0.00	0.00	0.00	0.00	0.00	0.00
	**Alcohols**		**17.10**	**0.09**	**1.15**	**8.24**	**3.46**	**2.18**	**5.91**	**0.25**	**0.00**	**0.00**	**0.09**
99	2-Pentanol	730	0.00	0.00	0.00	0.00	0.02	0.05	0.00	0.00	0.00	0.00	0.00
100	3-Methyl-1-Butanol	732	0.12	0.00	0.00	0.00	0.00	0.00	0.61	0.00	0.00	0.00	0.00
101	1-Pentanol	764	0.07	0.00	0.07	0.08	0.10	0.00	0.00	0.00	0.00	0.00	0.00
102	2,3-Butandiol	789	0.00	0.00	0.00	0.00	1.37	1.40	0.93	0.00	0.00	0.00	0.00
103	(*Z*)-3-Hexen-1-ol	854	0.16	0.02	0.01	0.00	0.00	0.00	0.00	0.00	0.00	0.00	0.00
104	(*E*)-2-Hexen-1-ol	866	0.75	0.04	0.11	0.00	0.00	0.00	0.00	0.00	0.00	0.00	0.00
105	1-Hexanol	869	14.37	0.03	0.35	0.00	0.00	0.00	1.14	0.25	0.00	0.00	0.00
106	1-Heptanol	970	0.23	0.00	0.00	0.00	0.00	0.00	0.00	0.00	0.00	0.00	0.00
107	1-Octen-3-ol	979	0.00	0.00	0.04	6.42	0.88	0.30	0.26	0.00	0.00	0.00	0.00
108	Benzyl alcohol	1030	0.00	0.00	0.13	0.00	0.28	0.21	0.23	0.00	0.00	0.00	0.00
109	(*Z*)-2-Octen-1-ol	1066	0.03	0.00	0.04	1.10	0.36	0.00	0.00	0.00	0.00	0.00	0.00
110	1-Octanol	1070	1.06	0.00	0.00	0.00	0.00	0.00	0.00	0.00	0.00	0.00	0.00
111	Phenyl ethyl alcohol	1105	0.00	0.00	0.00	0.00	0.00	0.00	2.42	0.00	0.00	0.00	0.09
112	1-Nonanol	1167	0.22	0.00	0.17	0.31	0.24	0.13	0.10	0.00	0.00	0.00	0.00
113	(*Z*)-2,6-octadien-1-ol 3,7dimethyl	1222	0.00	0.00	0.23	0.33	0.21	0.09	0.00	0.00	0.00	0.00	0.00
114	3-phenylpropanol	1222	0.09	0.00	0.00	0.00	0.00	0.00	0.22	0.00	0.00	0.00	0.00
	**Acids**		**0.12**	**0.48**	**0.58**	**0.13**	**3.48**	**8.79**	**2.51**	**11.54**	**3.48**	**32.03**	**21.89**
115	Acetic acid	604	0.06	0.21	0.00	0.00	2.15	7.83	1.69	9.98	3.48	0.00	3.63
116	2-Methyl butanoic acid	861	0.04	0.00	0.03	0.00	0.44	0.19	0.09	0.00	0.00	1.23	0.00
117	Hexanoic acid	997	0.02	0.27	0.42	0.02	0.89	0.20	0.64	1.56	0.00	19.96	17.59
118	Octanoic acid	1170	0.00	0.00	0.13	0.11	0.00	0.57	0.09	0.00	0.00	10.84	0.67
	**Alkanes**		**0.00**	**0.00**	**0.74**	**6.01**	**5.78**	**1.53**	**2.36**	**0.00**	**0.00**	**0.00**	**0.00**
119	2,3-Dimethyl hexane	761	0.00	0.00	0.00	0.03	0.00	0.00	0.00	0.00	0.00	0.00	0.00
120	Octane	820	0.00	0.00	0.00	0.03	0.00	0.00	0.00	0.00	0.00	0.00	0.00
121	4-Methyl octane	861	0.00	0.00	0.00	0.05	0.00	0.00	0.00	0.00	0.00	0.00	0.00
122	Nonane	900	0.00	0.00	0.05	0.15	0.00	0.00	0.00	0.00	0.00	0.00	0.00
123	Decane	998	0.00	0.00	0.00	0.11	0.00	0.00	0.00	0.00	0.00	0.00	0.00
124	Octyl cyclopropane	1069	0.00	0.00	0.43	0.00	1.20	0.38	0.00	0.00	0.00	0.00	0.00
125	Dodecane	1195	0.00	0.00	0.00	2.72	2.87	0.69	0.98	0.00	0.00	0.00	0.00
126	Tetradecane	1391	0.00	0.00	0.07	2.37	1.59	0.39	1.28	0.00	0.00	0.00	0.00
127	Hexadecane	1593	0.00	0.00	0.14	0.55	0.10	0.06	0.10	0.00	0.00	0.00	0.00
128	Heptadecane	1695	0.00	0.00	0.05	0.00	0.02	0.01	0.00	0.00	0.00	0.00	0.00
	**Terpenoids**		**18.28**	**41.57**	**39.89**	**23.32**	**19.48**	**16.14**	**15.14**	**53.31**	**79.24**	**30.31**	**26.86**
129	Sabinen	971	0.00	0.02	0.00	0.00	0.00	0.00	0.00	0.00	0.00	0.00	0.00
130	*β*-Myrcene	989	0.00	0.12	0.00	0.00	0.00	0.00	0.00	0.00	0.47	0.00	0.00
131	*d*-Limonene	1024	0.03	0.00	0.51	0.23	0.29	0.16	0.07	0.19	58.05	0.00	0.15
132	*β*-Ocimene	1046	0.02	0.06	0.24	0.17	0.17	0.05	0.00	0.00	0.00	0.00	0.00
133	Terpinolene	1083	0.02	0.15	0.31	0.00	0.00	0.00	0.00	0.00	0.00	0.00	0.00
134	*cis*-Linalol oxide	1084	0.00	0.00	0.00	0.00	0.45	0.00	0.00	0.00	0.00	0.00	0.83
135	Linalol	1096	11.96	2.30	16.43	7.15	6.99	2.46	4.40	0.70	0.00	5.18	0.49
136	Terpinen-4-ol	1172	0.00	0.00	0.00	0.00	0.14	0.00	0.00	0.00	0.00	0.00	0.00
137	*α*-Terpineol	1183	0.24	0.05	1.72	2.10	1.45	0.57	0.35	3.46	7.98	8.75	2.09
138	Myrtenol	1188	0.67	0.00	0.12	0.00	0.00	0.00	0.00	0.00	0.00	0.00	0.00
139	Geraniol	1248	0.01	0.00	0.55	0.00	0.00	0.00	0.00	0.00	0.00	0.00	0.00
140	Myrtenyl acetate	1317	0.07	0.00	0.00	0.00	0.00	0.19	0.00	0.00	0.00	0.00	0.00
141	*α*-Cubebene	1366	0.02	0.00	0.00	0.00	0.00	0.00	0.00	0.00	0.38	0.00	0.00
142	Caryophyllene	1408	0.02	0.00	0.00	0.00	0.00	0.00	0.00	0.00	0.24	0.00	0.00
143	*trans*-Geranyl acetone	1445	0.00	0.04	0.50	1.30	0.26	0.82	0.15	0.00	0.00	0.00	0.00
144	*cis*-β-Farnesene	1450	0.05	1.50	0.77	0.23	0.17	0.32	0.40	0.00	0.00	0.00	0.23
145	*α*-Curcumene	1476	0.00	0.04	0.05	0.00	0.00	0.00	0.00	0.00	0.00	0.00	0.00
146	(*Z,E)-α*-Farnesene	1489	0.00	0.33	0.22	0.00	0.07	0.07	0.00	0.00	0.00	0.00	0.00
147	*α*-Muurolene	1493	0.02	0.00	0.34	0.00	0.00	0.00	0.00	0.00	0.00	0.00	0.00
148	*α*-Farnesene	1500	0.01	1.10	0.50	0.35	0.29	0.47	0.07	0.00	0.00	0.00	0.00
149	(*Z*)-*γ*-Bisabolene	1508	0.00	0.07	0.05	0.00	0.00	0.00	0.00	0.00	0.00	0.00	0.00
150	*δ*-Cadenine	1519	0.00	0.00	0.25	0.00	0.00	0.00	0.00	0.00	0.00	0.00	0.00
151	*β*-Sesquiphellandrene	1519	0.00	0.04	0.00	0.00	0.00	0.00	0.00	0.00	0.00	0.00	0.00
152	(*E*)-*γ*-Bisabolene	1529	0.00	0.05	0.04	0.00	0.00	0.00	0.00	0.00	0.00	0.00	0.00
153	(*E*)-*α*-Bisabolene	1542	0.00	0.13	0.09	0.00	0.00	0.00	0.00	0.00	0.00	0.00	0.25
154	Myrtenyl-2-methyl butyrate	1556	0.00	0.08	0.00	0.00	0.00	0.00	0.00	0.00	0.00	0.00	0.00
155	(*E*)-Nerolidol	1563	5.13	31.17	17.14	11.04	8.97	10.78	9.65	40.99	9.94	13.75	17.92
156	Bisabolol oxide II	1655	0.00	0.03	0.06	0.62	0.15	0.06	0.04	5.32	1.51	2.00	3.27
157	*α*-Bisabolol	1683	0.01	3.97	0.00	0.11	0.08	0.09	0.00	2.65	0.67	0.63	1.63
158	Nerolidyl acetate	1711	0.00	0.25	0.00	0.02	0.00	0.10	0.01	0.00	0.00	0.00	0.00
159	Farnesol	1720	0.00	0.07	0.00	0.00	0.00	0.00	0.00	0.00	0.00	0.00	0.00
	**Others**		**14.56**	**0.33**	**5.14**	**4.53**	**4.17**	**1.39**	**1.72**	**0.00**	**2.09**	**0.22**	**0.00**
160	Styrene	887	14.29	0.02	0.05	0.00	0.00	0.00	0.00	0.00	2.09	0.22	0.00
161	2-Pentyl furan	990	0.24	0.00	0.00	0.00	0.00	0.00	0.00	0.00	0.00	0.00	0.00
162	Pyranone	1135	0.00	0.00	0.00	0.09	0.28	0.00	0.00	0.00	0.00	0.00	0.00
163	*m*-Di-*tert*-butyl benzene	1247	0.00	0.00	0.00	4.43	3.75	1.00	1.56	0.00	0.00	0.00	0.00
164	1-Methoxy-4-(1-propenyl) benzene	1277	0.00	0.00	5.09	0.00	0.00	0.00	0.00	0.00	0.00	0.00	0.00
165	Sulfur	2049	0.03	0.31	0.00	0.01	0.14	0.39	0.16	0.00	0.00	0.00	0.00
	**Total identified**		**92.79**	**94.81**	**96.88**	**96.85**	**89.66**	**95.24**	**95.88**	**85.03**	**86.81**	**95.14**	**91.96**

Jam 1 (Coop Vivi verde jam); Jam 2 (Coop jam); Jam 3 (Zuegg jam); Jam 4 (Alce Nero jam).

**Table 2:** Ingredients listed on the label of each strawberry jam used in this study.

Jam	Ingredients (100 g Jam Product)
Jam 1 (Coop Vivi verde jam)	60 g strawberry, sugar, lemon juice, pectin, citric acid
Jam 2 (Coop jam)	50 g strawberry, sugar, lemon juice, pectin, citric acid, elderberry juice
Jam 3 (Zuegg jam)	50 g strawberry, sugar, lemon juice, pectin
Jam 4 (Alce Nero jam)	100 g strawberry, lemon juice, grapes juice

## Data Availability

Not applicable.

## Sample Availability

The samples of dried plant materials are available from authors upon the reasonable request.
